# Global Epidemiology of Human Adenoviruses, 2016–2024: A Pre‐ and Post‐COVID‐19 Analysis of Circulation Patterns and Epidemic Timing

**DOI:** 10.1111/irv.70236

**Published:** 2026-03-04

**Authors:** Emma Papini, Guglielmo Bonaccorsi, Angela Bechini, Fabiola Berti, Sara Boccalini, Paolo Bonanni, Manuela Chiavarini, Claudia Cosma, Chiara Lorini, Cristina Salvati, Valentina Saviozzi, Patrizio Zanobini, Saverio Caini, Marco Del Riccio

**Affiliations:** ^1^ Department of Health Sciences University of Florence Florence Italy; ^2^ University of Florence Florence Italy

**Keywords:** COVID‐19 pandemic, Human adenovirus, respiratory viruses, seasonality, virological surveillance

## Abstract

**Background:**

Human adenoviruses (HAdV) circulate globally, but their seasonal patterns remain poorly defined. We aimed to characterize the timing, amplitude, and duration of HAdV epidemics worldwide and to compare patterns before and after the COVID‐19 pandemic.

**Methods:**

Virological surveillance data on HAdV were obtained from the WHO FluNet database: data from 65 countries were analyzed to estimate epidemic peak timing, amplitude, and duration across the Northern and Southern Hemispheres and the intertropical belt, comparing prepandemic (2016–2019) with postpandemic (2021–2024) periods. To ensure robustness, analyses were restricted to country‐seasons with ≥ 30 reporting weeks.

**Results:**

From 2016 to 2024, 65 countries reported roughly 148,000 HAdV detections across 335 country‐seasons; 46% of seasons had ≥ 50 detections. In the 20 countries with sufficient data for seasonality analyses, median epidemic duration was 31 weeks (range 5–42) and median peak amplitude 70% (40%–98%). Peak timing followed latitude: June–July in Southern Hemisphere, November–December in high‐latitude Northern countries, March–April in lower latitude. After COVID‐19, several countries showed marked timing shifts, with concurrent changes in amplitude.

**Conclusions:**

After the onset of the COVID‐19 pandemic, the usual seasonal patterns of HAdV were altered, with pronounced shifts in peak timing across settings and latitudes. These results underscore the need for strong, ongoing, type‐specific surveillance to guide public health strategies.

## 1 Introduction

Human adenoviruses (HAdV) (genus *Mastadenovirus*, family Adenoviridae) are double‐stranded DNA, nonenveloped icosahedral virions, which make them remarkably resistant to detergents, acidic conditions and moderate heating [1]. There are seven HAdV subgroups recognized to date (HAdV‐A–G), and they are further classified in approximately 51 serotypes and 116 unique genotypes [2, 3, 4]. Different serotypes display specific tissue tropisms closely mirroring their clinical presentations: For instance, HAdV‐B serotypes 3 and 7 tend to infect the lower respiratory tract and often precipitate severe pneumonia, whereas species F serotypes 40 and 41 target enterocytes and are established causes of pediatric gastroenteritis [2].

Even if most infections are subclinical, HAdV can produce a broad clinical spectrum that ranges from self‐limited conjunctivitis to life‐threatening multisystem disease in immunocompromised hosts, where they may present as fulminant pneumonia, hepatitis, hemorrhagic cystitis, or disseminated multiorgan infection [1, 5, 6]. Transmission is predominantly directly from one infected individual to another, via inhalation of aerosolized droplets, direct conjunctival inoculation or via fecal‐oral spread [7, 8]. Respiratory droplets from coughs and sneezes, together with contaminated hands or objects such as environmental surfaces, towels, infected tissue, toys and utensils, are common vehicles [8]. After an incubation period of about 2–14 days, infection may become symptomatic or remain silent; HAdV can establish long‐term latency in lymphoid tissue, renal parenchyma, and other organs. In immunosuppressed hosts, they can reactivate and cause disseminated disease [9, 10]. Even in immunocompetent individuals, asymptomatic shedding may continue for weeks to months, maintaining a reservoir for onward spread [10]. In fact, after apparent recovery, infected individuals can continue to shed, often without symptoms [7]. Because of their resistance to most common disinfectants, environmental persistence, and prolonged postinfection shedding, HAdV can circulate continuously within communities [8, 11].

In contrast to several other respiratory viruses, the seasonality of HAdV remains uncertain. While occasional winter peaks have been described in small local surveys, surveillance studies indicate that increases in detections can occur in any month and across different continents [1, 6, 12, 13, 14, 15, 16]. Seasonal peaks varied by latitude before the pandemic and shifted modestly afterward across all regions [14]. HAdV epidemics are most often reported in semiclosed or closed settings such as military barracks, long‐term care institutions, and day‐care centers [10]; in this context, a live oral vaccine against HAdV Types 4 and 7, which has markedly reduced respiratory outbreaks in training facilities, continues to be administered routinely to US military recruits [17].

Accurate estimates of incidence remain difficult because HAdV infection is not a notifiable disease in many countries [18]. Nevertheless, unlike seasonal respiratory viruses such as influenza, HAdV appear to circulate year‐round and do not show a clearly consistent annual pattern [8]. Given the scarcity of global data on HAdV circulation, we analyzed WHO FluNet surveillance data from 2016 to 2025 (i.e., including both pre‐ and post‐COVID‐19 pandemic data) to characterize worldwide patterns, comparing regions and defining epidemic timing, peak intensity, and duration.

## 2 Methods

### 2.1 Data Sources Definitions

Weekly counts of laboratory‐confirmed HAdV detections were extracted from the WHO FluNet database (https://www.who.int/tools/flunet) on July 15, 2025, which ensured data availability up to epidemiological Week 26 of 2025 [19]. Each country was allocated to a latitudinal area according to the geographic centroid, namely, the Northern hemisphere (NH), north of the Tropic of Cancer; the Southern hemisphere (SH), south of the Tropic of Capricorn; and the intertropical belt (ITB), between the two tropics. Owing to the opposite timing of respiratory‐virus activity across latitudes, a “season” was defined as the calendar year (Weeks 01–52/53) for ITB and SH countries and as the period from Week 27 of a given calendar year to Week 26 of next year for NH countries [14]. According to this definition, using surveillance data from Week 1/2016 to Week 26/2025 ensured that up to nine full seasons could be available for countries in both the NH (from 2016–2017 to 2024–2025) and the ITB and SH (from 2016 to 2024). Of note, Weeks 1–26/2016 in the NH, and Weeks 1–26/2025 in the ITB and SH were not used in the analysis, as they would correspond to half a season in those geographical areas. The unit of analysis in the present investigation is the “country‐season”, i.e., data for a “season” (defined as above) in a given country.

Within FluNet, it is currently not possible to calculate a positivity rate for HAdV due to the lack of the denominator for this calculation (i.e., the number of specimens actually used for HAdV testing). For this reason, this investigation was mostly focused on the seasonality of HAdV epidemics, defined both in terms of peak timing and duration of epidemics.

In the WHO FluNet database, each country can contribute data labelled as follows:

- “Sentinel surveillance”: data gathered regularly and systematically within sentinel surveillance systems
- “Nonsentinel surveillance”: data originating from outbreak investigations, universal testing, point‐of‐care testing, or other testing systems apart from surveillance
- “Not defined”: data lacking a specific categorization (possibly including combined sentinel and nonsentinel data).

For some countries, data for a given season were available that originated from more than one type of surveillance system. Unlike the positivity rate, there is no a priori reason to believe that the timing and duration of epidemics differ for HAdV cases of different severity (the latter being a main determinant of a case being seen in different types of surveillance systems). Based on this assumption, we chose to use, for each country‐season, data from the surveillance system type with the highest number of HAdV detections, in order to enhance statistical power and robustness of epidemic timing estimation. Prior to this, country‐seasons with fewer than 30 weeks of data were removed from the dataset, regardless of the number of HAdV detections and of the type of surveillance system, because of the possibility that they might not cover the entire epidemic and therefore provide a biased value of the epidemic peak timing and duration.

### 2.2 Statistical Analysis

We extracted the number of HAdV detections reported to FluNet in country‐season and classified these into three classes depending on whether there were 1–24, 25–49, or ≥ 50 detections (country‐seasons with 0 detections were excluded because the absence of testing could not be distinguished from the lack of detections). We then described the distribution of country‐seasons by category of HAdV detections reporting according to latitudinal zone, WHO region, and season [19].

We studied the seasonality of HAdV circulation by analyzing country‐specific time series using the EPIPOI software (https://www.epipoi.info/) [20, 21]. The season 2020 (2020–2021 for countries in the NH) was not included in the seasonality analysis because it was particularly atypical due to the COVID‐19 pandemic. Our objective was to describe the global seasonality of HAdV epidemics, also comparing the period before the COVID‐19 pandemic with the postpandemic period. To ensure robustness of the results, we included in the seasonality analyses only those countries that had at least three seasons with 50+ reported HAdV detections in either the prepandemic or postpandemic period. EPIPOI operates by first detrending the country‐specific time series using a quadratic polynomial and then working out the periodic annual function (PAF) of the time series by summing up the annual, semiannual, and quarterly harmonics (obtained via Fourier decomposition). The typical timing of the peak corresponds to the peak month of the PAF, while the amplitude is obtained as the ratio of the wave height to the peak value and is expressed as a percentage. The peak amplitude can be interpreted as an estimate of the degree to which cases cluster around the typical peak timing; due to how it is calculated, the amplitude can sometimes exceed 100% [21]. The timing and amplitude of the primary peak were first calculated for every country using data from the entire study window (2016–2025), and then separately for the prepandemic and postpandemic periods for countries that had at least three seasons with 50+ HAdV detections in both periods.

Finally, the duration of the HAdV epidemic was calculated in each country‐season using the 75% Average Annual Percentage (AAP) method, i.e., by defining the shortest consecutive run of weeks that accounted for at least 75% of overall detections in that country‐season.

### 2.3 Software

All analyses were conducted using Stata version 17 (Stata Corp, College Station, TX, USA) and the freely available analytical software EPIPOI (https://www.epipoi.info/) [20].

## 3 Results

### 3.1 Data Availability and Descriptive Summary

Between 2016 and 2024, 65 countries and territories reported HAdV detections to FluNet, providing ~148,000 detections across 335 country‐seasons. The contribution of individual countries was heterogeneous: The median number of detections per country‐season was 37, ranging from fewer than 25 detections in about 40% of seasons to ≥ 50 detections in almost half (Table S1). Annual total detections fluctuated markedly: A minimum was recorded in 2020 (just over 5000 detections), followed by a progressive increase in 2022–2024, when the number of detections and the proportion of country‐seasons with ≥ 50 cases reached their highest levels. Median values per country‐season followed the same pattern, increasing from 13 in 2020 to 95 in 2024. When grouped by latitude and WHO region, the distribution of detections again showed substantial heterogeneity: higher totals were generally reported from countries in the Americas and Western Pacific, consistent with the contributions given by individual countries, while data availability remained limited in parts of Africa and were absent for Europe in this dataset (Tables [Link], [Link]). These differences should be interpreted cautiously, as they largely mirror the uneven distribution of surveillance capacity.

A summary of detections by latitude, WHO region, and season is shown in Tables [Link], [Link]; country‐specific time series for the study period are also provided in Figures [Link], [Link]. Only country‐seasons with ≥ 50 detections were included.

### 3.2 Peak Timing and Duration of HAdV Epidemics

Considering the cutoffs applied and previously described in the methods section, the primary seasonal peak and its characteristics (month of peak, amplitude, and duration) could be summarized for 20 of all 65 countries reporting at least one detection in the considered period. Across these countries, the median epidemic duration was 31 weeks (country‐level medians), with a global range from 5 to 42 weeks (Table 1).

**TABLE 1:** Month, amplitude, and duration of the primary seasonal HAdV peak by country, with global values and period‐specific ranges (2016–2024).

Country	Hemisphere	Latitude	2016–2024			
			N. seasons with ≥ 50 reported HAdV cases included in the analysis of seasonality	Duration in weeks (75% AAP), range (median)	Month primary peak	Amplitude primary peak (%)
**Chile**	**Southern**	−37.7	8(2016–2019, 2021–2024)	17 to 36 (29)	5.4 (Jun)	70.8%
**Argentina**	**Southern**	−35.4	8 (2016–2019, 2021–2024)	27 to 36 (31)	6.2 (Jul)	74.3%
**Australia**	**Southern**	−25.7	8 (2016–2019, 2021–2024)	20 to 33 (31)	6.8 (Jul)	58.4%
**Paraguay**	**ITB**	−23.2	6 (2016, 2018–2019, 2022–2024)	22 to 37 (25.5)	7.5 (Aug)	85.0%
**Brazil**	**ITB**	−10.8	6 (2017–2019, 2021, 2023–2024)	25 to 32 (30)	8.9 (Sep)	78.4%
**Colombia**	**ITB**	3.9	7 (2016–2019, 2022–2024)	19 to 39 (36)	10.4 (Nov)	60.4%
**Panama**	**ITB**	8.5	7 (2016–2019, 2022–2024)	26 to 33 (31)	6.0 (Jun–Jul)	69.0%
**Costa Rica**	**ITB**	10.0	7 (2017–2019, 2021–2024)	13 to 35 (32)	2.7 (Mar)	75.4%
**Thailand**	**ITB**	15.1	3 (2022–2024)	19 to 40 (34)	3.1 (Apr)	90.6%
**Guatemala**	**ITB**	15.7	5 (2017–2019, 2022–2023)	22 to 30 (27)	5.7 (Jun)	76.8%
**Dominican Republic**	**ITB**	18.9	3 (2022–2024)	17 to 40 (18)	4.9 (May)	89.9%
**Oman**	**ITB**	20.6	7 (2017–2019, 2021–2024)	25 to 38 (36)	1.6 (Feb)	40.0%
**Hong Kong**	**ITB**	22.4	7 (2016–2019, 2022–2024)	5 to 42 (33.5)	6.4 (Jul)	40.6%
**India**	**ITB**	22.9	5 (2017–2018, 2022–2024)	19 to 39 (33)	2.1 (Mar)	70.4%
**Mexico**	**Northern**	23.9	5 (2018, 2021–2024)	31 to 34 (34)	2.2 (Mar)	67.5%
**United Arab Emirates**	**Northern**	23.9	4 (2021–2024)	26 to 37 (29.5)	3.3 (Apr)	66.3%
**Qatar**	**Northern**	25.3	8 (2016–2019, 2021–2024)	23 to 36 (30)	3.5 (Apr)	64.9%
**Japan**	**Northern**	37.6	8 (2016–2019, 2021–2024)	30 to 40 (35)	5.7 (Jun)	56.4%
**Mongolia**	**Northern**	46.8	4 (2019, 2021–2023)	19 to 34 (25.5)	11.0 (Nov–Dec)	97.9%
**Canada**	**Northern**	61.4	8 (2016–2019, 2021–2024)	29 to 38 (33)	11.7 (Dec)	68.5%

**FIGURE 1:**
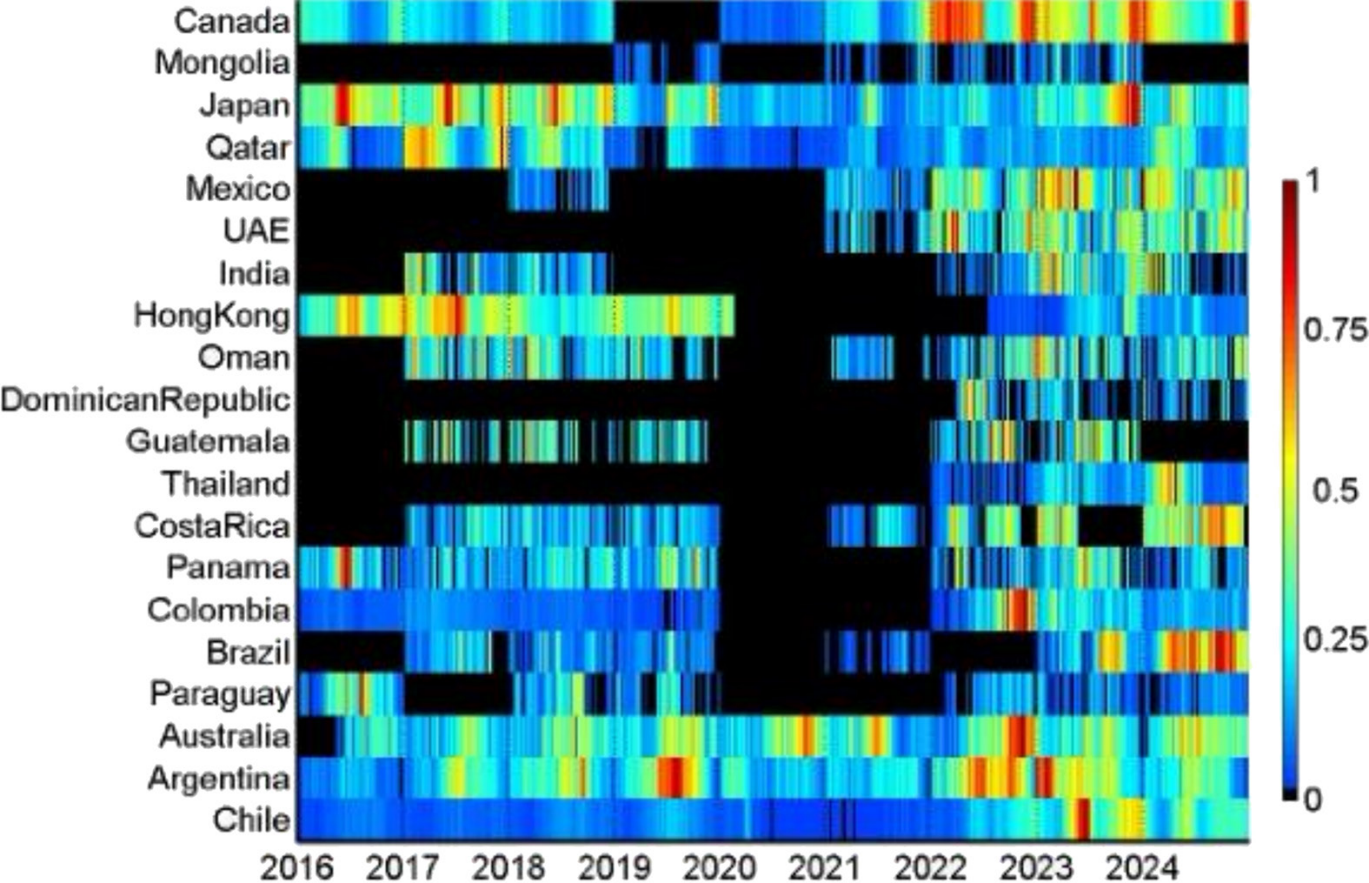
Heatmap of HAdV detections by country and month. Note: colors represent the relative monthly intensity of detections within each country and period, scaled from low (blue) to high (red).

**FIGURE 2:**
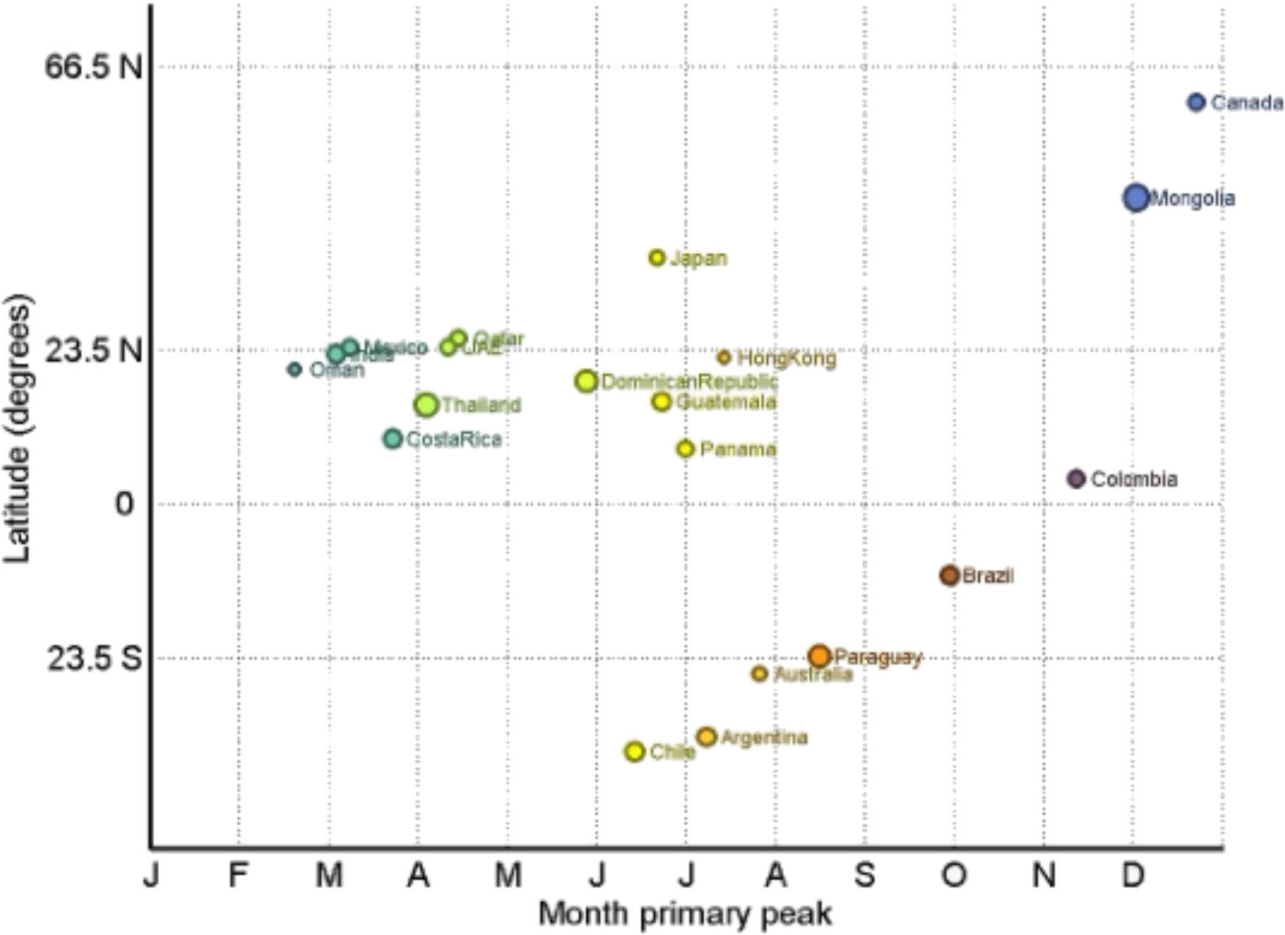
Timing of the primary seasonal HAdV peak by country latitude, global analysis, 2016–2024. Note: Colors vary depending on each country's primary peak timing.

Timing patterns differed systematically by latitude: In SH countries, peaks clustered in midyear: Chile, Argentina, and Australia peaked in June–July (median peak month across these countries 6.2). In NH countries, two timing tendencies were evident: high‐latitude countries peaked in late autumn/early winter (e.g., Mongolia in November–December, or Canada in December), while lower latitude Northern settings peaked earlier in the year (Mexico in March; Qatar and the United Arab Emirates in April; Japan in June). In the **ITB**, peaks were more dispersed across the calendar, as shown in the plot (Figures 1, 2).

Out of the 20 analyzed countries, 13 had at least three seasons with 50+ HAdV detections in both periods, and therefore, a comparison between pre‐ and post‐2020 was possible; as shown in Table 2, some countries differed markedly in peak timing between the pre‐COVID period (2016–2019) and the period following the emergence of COVID‐19 (2021 onwards). The largest shifts were observed in Colombia (from an April peak to a November peak), Costa Rica (from an end‐September peak to a March peak), and in Hong Kong and Japan (from a June–July peak to a November–December peak) (Table 2).

**TABLE 2:** Comparison of HAdV epidemic timing, duration, and amplitude before and after the COVID‐19 pandemic (selected countries, 2016–2019 vs. 2021–2024).

Country	Hemisphere	Latitude	2016–2019			2021–2024		
			Duration in weeks (75% AAP), range	Month primary peak	Amplitude primary peak (%)	Duration in weeks (75% AAP), range	Month primary peak	Amplitude primary peak (%)
**Chile**	Southern	−37.7	28 to 33	6.7 (Jul)	71.0%	22 to 36	5.4 (Jun)	79.1%
**Argentina**	Southern	−35.4	27 to 32	6.8 (Jul)	89.0%	30 to 35	5.7 (Jun)	67.5%
**Australia**	Southern	−25.7	20 to 33	7.7 (Aug)	65.2%	25 to 33	6.3 (Jul)	60.4%
**Paraguay**	ITB	−23.2	24 to 27	7.4 (Aug)	91.0%	22 to 37	8.6 (Sep)	70.6%
**Brazil**	ITB	−10.8	26 to 31	6.2 (Jul)	80.8%	25 to 32	9.0 (Sep‐Oct)	80.6%
**Colombia**	ITB	3.9	35 to 39	3.7 (Apr)	30.1%	19 to 36	10.4 (Nov)	60.9%
**Panama**	ITB	8.5	26 to 33	6.0 (Jun–Jul)	75.5%	27 to 33	6.1 (Jul)	64.7%
**Costa Rica**	ITB	10.0	32 to 34	9.0 (Sep–Oct)	61.9%	15 to 35	2.8 (Mar)	87.0%
**Oman**	ITB	20.6	36 to 38	1.9 (Feb)	74.9%	25 to 36	0.1 (Jan)	49.7%
**Hong Kong**	ITB	22.4	33 to 42	6.4 (Jul)	41.8%	15 to 34	11.1 (Dec)	82.6%
**Qatar**	Northern	25.3	23 to 36	3.9 (Apr)	64.0%	30 to 36	3.1 (Apr)	67.7%
**Japan**	Northern	37.6	30 to 40	6.0 (Jun–Jul)	55.7%	32 to 35	11.0 (Nov–Dec)	60.9%
**Canada**	Northern	61.4	29 to 34	11.4 (Dec)	68.2%	32 to 38	11.9 (Dec)	71.1%

**FIGURE 3:**
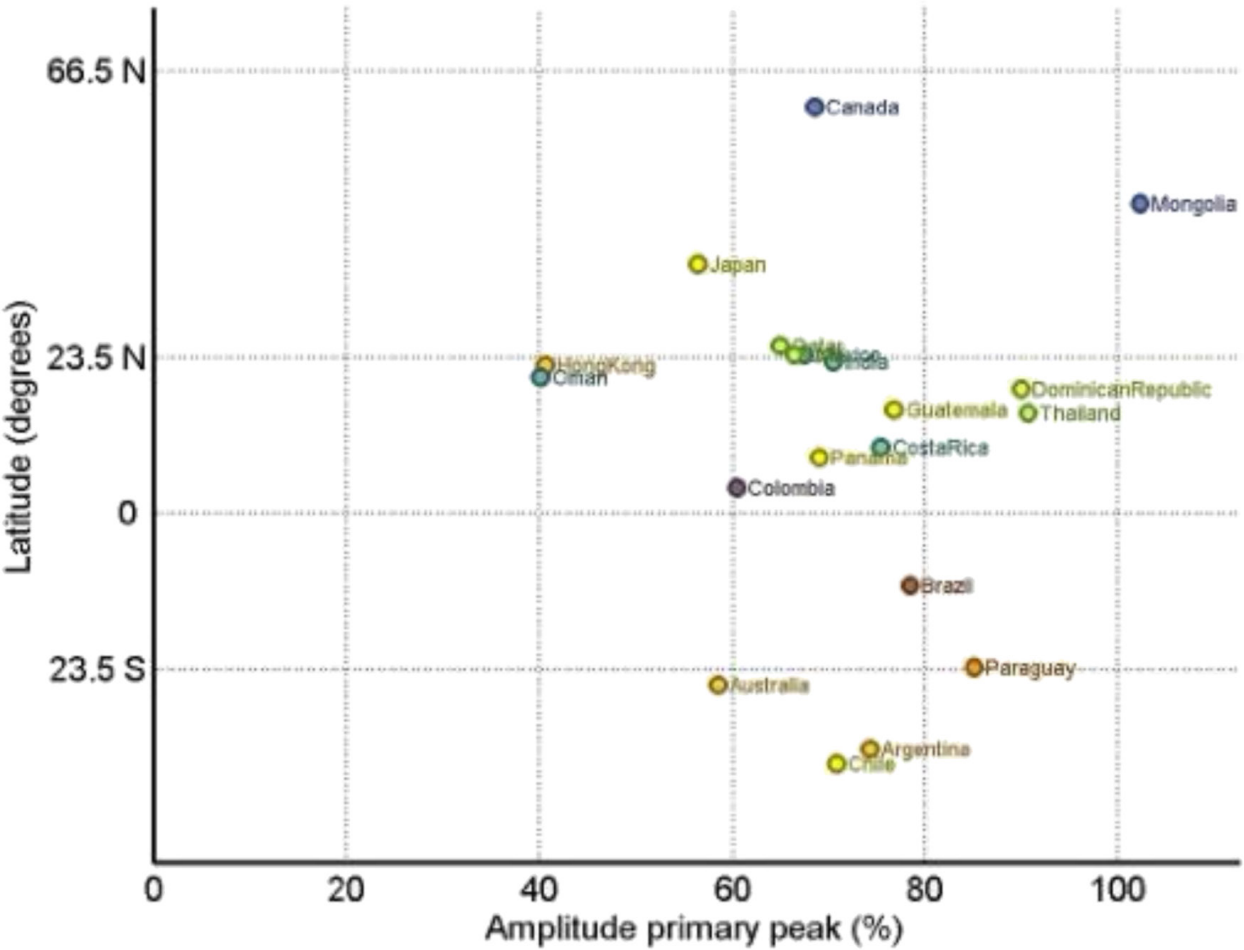
Relationship between the amplitude of the primary seasonal HAdV peak in a given country and country latitude. Note: Colors vary depending on each country's primary peak timing.

The amplitude of the primary peak varied widely across different countries, with a global median of 69.7% and values spanning from 40.0% to 97.9% across countries (Table 1 and Figure 3).

Low‐amplitude patterns, indicating a less pronounced seasonal peak relative to the annual baseline, were observed in countries such as Oman and Hong Kong; in contrast, high‐amplitude patterns (often coupled with shorter durations) were observed in Mongolia, the Dominican Republic, Thailand, Paraguay, and Brazil. Most Southern temperate countries exhibited moderate‐to‐high amplitudes, with Australia as a low‐amplitude outlier. In countries with data for both periods pre‐ and post‐COVID, median amplitude increased in the NH (64.0% to 67.7%) and decreased in the SH (71.0% to 67.5%) and in the ITB (74.9% to 70.6%).

*Duration of the epidemics*: country‐level median durations ranged from 18 weeks (Dominican Republic) to 36 weeks (Colombia and Oman), with an overall median of 31 weeks. The shortest documented lower bound of the duration range was 5 weeks and the longest upper bound 42 weeks, both in Hong Kong, indicating the widest within‐country spread. By area, median durations were similar, with 32 weeks in the ITB (range across countries 18–36), 31.5 weeks in the NH (25.5–35), and 31 weeks in the SH (29–31). These distributions are consistent with the visual impression of broader, lower amplitude activity in intertropical settings and more concentrated midyear peaks in Southern temperate countries shown in Figure 1.

## 4 Discussion

To our knowledge, this is the first global analysis to characterize the timing, amplitude, and length of HAdV circulation across years and locations using WHO FluNet surveillance data. Our evidence indicates that HAdV can be best viewed as year‐round viruses, exhibiting sustained circulation in most circumstances and only partial overlap with the seasonal patterns of other respiratory pathogens. The analysis shows a latitudinal gradient for peak timing, with midyear peaks for HAdV in the SH and end‐year peaks in NH, a general common pattern for respiratory viruses; however, for comparison with influenza and respiratory syncytial virus (RSV), for which the epidemic duration is largely confined to 8–16 weeks [14, 22, 23], HAdV epidemics in our dataset had substantially longer duration with a median period of approximately 31 weeks and maxima of 36 weeks despite some exceptions with shorter durations. Moreover, unlike influenza, which typically peaks in midwinter, HAdV peaks occurred in late autumn–early winter in most temperate countries (either SH or NH countries). In tropical and subtropical settings, peak months were more irregularly distributed throughout the year, not unlike influenza and other respiratory viruses. Across the study period, clear disruptions in the timing and intensity of peaks were observed right after the COVID‐19 pandemic, but the overall pattern tended to re‐emerge in subsequent years [14].

Across the considered time period, detection volume increased substantially after 2020 (excluding 2020–2021), peaking in 2023 and still remaining high in 2024, and the percentage of seasons in which at least 50 detections occurred followed a similar trajectory. These patterns, while indicative of the disruption caused by the COVID‐19 pandemic and the subsequent resumption of social contact, were also likely affected by the strengthening of surveillance systems, enhanced awareness of healthcare providers, and increased use in multiplex diagnostic panels, which heighten the probability of detecting HAdV in clinical/practice and laboratory environments [24, 25, 26, 27]. Thus, it is wise to interpret inter annual variability as more than an expression of inherent epidemiological fluctuations but rather the effect of enhancements and changes in the ability to diagnose and reporting systems [3, 18].

From an epidemiological perspective, the contrast between temperate and tropical settings is clear but not binary; in temperate countries, peaks aligned with winter‐adjacent months, yet epidemics remained protracted relative to influenza/RSV, underscoring differences in transmission dynamics [14, 22]. In the tropics and subtropics, we have weaker or multimodal climatic forcing, which also aligns with flatter or irregular peaks and reflects wider evidence regarding respiratory virus seasonality and meteorological drivers [12, 13, 16]. Observed local deviations from hemispherical patterns, such as peak month preceding versus post‐2020, are likely a combination of altered contact patterns, population immunity dynamics/modelling, and testing behaviors observed during the pandemic period [3, 14].

Several biological characteristics possibly account for the prolonged circulation patterns of HAdV activity; HAdV can persist and reactivate in certain tissues, and asymptomatic shedding can occur for weeks to months, which could establish reservoirs for onward transmission [8, 10, 28]. Their nonenveloped capsids enable them to persist in the environment, spread indirectly, and remain viable on fomites, characteristics associated with outbreaks of keratoconjunctivitis and respiratory disease in community and healthcare settings [1, 8, 11]. Furthermore, the substantial diversity in species and tissue tropisms, encompassing respiratory, enteric, and ocular syndromes, extends the opportunities for clinical detection throughout the calendar year [5, 29]. These characteristics, in conjunction with climate variability, can lead to long and diffuse epidemic profiles across the calendar year even if peaks are showing latitude dependency.

The public health implications are immediate: Surveillance should be continuous rather than seasonal to detect shifts in the timing, intensity, and type distribution of HAdV infections. With the increasing use of multiplex assays, HAdV is now detected year‐round, but prolonged shedding complicates clinical attribution, and the burden appears diffuse across the year rather than concentrated in short, winter periods [8, 28]. Preparedness planning should recognize the temporally diffuse burden of HAdV infections, requiring sustained service delivery across pediatrics, respiratory care, and ophthalmology rather than seasonal surge capacity. Incorporating HAdV into type‐specific reporting systems, building on platforms such as GISRS/FluNet and national surveillance networks, would strengthen risk assessment and outbreak response. Moreover, the existing experience with live oral vaccines against HAdV‐4 and HAdV‐7 provides an operational framework to conceptualize future civilian vaccination strategies in higher risk settings [17].

This study has several limitations: data completeness and representativeness varied regionally: Europe provided no HAdV data, reporting from Africa and Southeast Asia was sparse, while the Americas and Western Pacific contributed disproportionately—reducing the generalizability of the global summaries. Moreover, the analyzed countries differed greatly in size and populations. Many laboratories are nested in influenza‐based case definitions and workflows, which may differentially sample HAdV and underrepresent or miss nonrespiratory presentations. We analyzed detections in their broadest sense and did not discriminate between species/genotype; it would preclude any analysis of type‐specific seasonal niches and severity profiles in spite of known biological heterogeneity [1, 8, 29]. We did not have information on environmental covariates, and we did not model viral–viral interactions, both of which could help inform timing and amplitude [12, 14, 16].

## 5 Conclusion

Our FluNet analysis reframes HAdV as a pathogen that has prolonged, diffuse epidemics and latitude related timing, disrupted in 2020–21 and partially re‐established subsequently; it stands in contrast with the short, winter‐accommodating waves of influenza/RSV and makes a case for surveillance that is continuous, type resolved, and integrated with environmental and interaction data [14, 22]. Updating and adopting existing infrastructures, such as FluNet/GISRS and national type reporting systems, would facilitate earlier shifts in disease detection and more calibrated diagnostics and service capabilities. Finally, high‐resolution, continuous genotyping is the critical missing layer to define specific risk groups and evaluate targeted vaccination in closed or high‐risk settings, where this type of impact has been demonstrated for HAdV 4/7 [17]. Following the initial global description, strengthening surveillance will be indispensable for translating broad signals into actionable insights, thereby enabling health systems to respond effectively to a pathogen characterized by constant, though often neglected, presence.

## Author Contributions

**E.P.:** conceptualization, methodology, data curation, formal analysis, writing – original draft. **G.B.:** writing – review and editing, validation. **A.B.:** writing – review and editing, validation. **F.B.:** writing – review and editing, validation. **S.B.:** writing – review and editing, validation. **P.B.:** writing – review and editing, validation. **M.C.:** writing – review and editing, validation. **C.C.:** writing – review and editing, validation. **C.L.:** writing – review and editing, validation. **C.S.:** writing – review and editing, validation. **V.S.:** writing – review and editing, validation. **P.Z.:** writing – review and editing, validation. **S.C.:** conceptualization, methodology, data curation, formal analysis, validation, writing – review and editing. **M.D.R.:** conceptualization, methodology, supervision, data curation, project administration, validation, writing – original draft, writing – review and editing.

## Ethics Statement

The authors have nothing to report.

## Conflicts of Interest

The authors declare no conflicts of interest.

## Supporting information


**Table S1:** Global circulation of HAdV in countries lying in the NH, ITB, or SH.

**Table S2:** Global circulation of HAdV by WHO region.

**Table S3:** Global circulation of HAdV by season from 2016 to 2024.

**Figure S4:** Time series of circulation by country.

**Figure S5:** Supporting information.

**Figure S6:** Supporting information.

**Figure S7:** Supporting information.

**Figure S8:** Supporting information.

**Figure S9:** Supporting information.

**Figure S10:** Supporting information.

**Figure S11:** Supporting information.

**Figure S12:** Supporting information.

**Figure S13:** Supporting information.

**Figure S14:** Supporting information.

**Figure S15:** Supporting information.

**Figure S16:** Supporting information.

**Figure S17:** Supporting information.

**Figure S18:** Supporting information.

**Figure S19:** Supporting information.

**Figure S20:** Supporting information.

**Figure S21:** Supporting information.

**Figure S22:** Supporting information.

**Figure S23:** Supporting information.

**Figure S24:** Supporting information.

## Data Availability

The data are publicly available and can be downloaded from the WHO FluNet website.
