# Supplementary material for: Global Epidemiology of Human Adenoviruses, 2016–2024: A Pre‐ and Post‐COVID‐19 Analysis of Circulation Patterns and Epidemic Timing
Source: Influenza Other Respir Viruses. 2026 Mar 4;20(3):e70236. doi: 10.1111/irv.70236 (PMC12959972; doi:10.1111/irv.70236)
Supplement: Supplementary file 1 — Table S1: Global circulation of HAdV in countries lying in the NH, ITB, or SH. [file IRV-20-e70236-s008.docx]

Supplementary Table S1: Global circulation of HAdV in countries lying in the NH, ITB, or SH

| **Geographical area** | **N. of HAdV detections reported to Flunet** | **Median detections per country-season** | **N. (%) country-seasons with 1-24 reported cases** | **N. (%) country-seasons with 25-49 reported cases** | **N. (%) country-seasons with ≥ 50 reported cases** |
| --- | --- | --- | --- | --- | --- |
| Northern hemisphere | 61,153 | 91 | 21 (28.4%) | 8 (10.8%) | 45 (60.8%) |
| Inter‑tropical belt | 39,966 | 23 | 116 (52.3%) | 26 (11.7%) | 80 (36.0%) |
| Southern hemisphere | 46,881 | 666 | 7 (17.9%) | 3 (7.7%) | 29 (74.4%) |
| **Total** | **148,000** | **37** | **144 (43.0%)** | **37 (11.0%)** | **154 (46.0%)** |
