# Supplementary material for: Global Epidemiology of Human Adenoviruses, 2016–2024: A Pre‐ and Post‐COVID‐19 Analysis of Circulation Patterns and Epidemic Timing
Source: Influenza Other Respir Viruses. 2026 Mar 4;20(3):e70236. doi: 10.1111/irv.70236 (PMC12959972; doi:10.1111/irv.70236)
Supplement: Supplementary file 2 — Table S2: Global circulation of HAdV by WHO region. [file IRV-20-e70236-s018.docx]

Supplementary Table S2: Global circulation of HAdV by WHO region

| **WHO region** | **N. of HAdV detections reported to Flunet** | **Median detections per country-season** | **N. (%) country-seasons with 1-24 reported cases** | **N. (%) country-seasons with 25-49 reported cases** | **N. (%) country-seasons with ≥ 50 reported cases** |
| --- | --- | --- | --- | --- | --- |
| African Region (AFR) | 445 | 14 | 10 (58.8%) | 4 (23.5%) | 3 (17.7%) |
| Region of the Americas (AMR) | 92,888 | 28 | 92 (48.2%) | 22 (11.5%) | 77 (40.3%) |
| Eastern Mediterranean (EMR) | 13,233 | 78 | 14 (32.5%) | 3 (7.0%) | 26 (60.5%) |
| European Region (EUR) | 0 | - | - | - | - |
| South‑East Asia (SEAR) | 1,946 | 25 | 10 (50.0%) | 2 (10.0%) | 8 (40.0%) |
| Western Pacific (WPR) | 39,488 | 100 | 18 (28.1%) | 6 (9.4%) | 40 (62.5%) |
| **Total** | **148,000** | **37** | **144 (43.0%)** | **37 (11.0%)** | **154 (46.0%)** |
