# Supplementary material for: Global Epidemiology of Human Adenoviruses, 2016–2024: A Pre‐ and Post‐COVID‐19 Analysis of Circulation Patterns and Epidemic Timing
Source: Influenza Other Respir Viruses. 2026 Mar 4;20(3):e70236. doi: 10.1111/irv.70236 (PMC12959972; doi:10.1111/irv.70236)
Supplement: Supplementary file 3 — Table S3: Global circulation of HAdV by season from 2016 to 2024. [file IRV-20-e70236-s013.docx]

Supplementary Table S3: Circulation of HAdV by season from 2016 to 2024

| **Season** | **N. of HAdV detections reported to Flunet** | **Median detections per country-season** | **N. (%) country-seasons with 1-24 reported cases** | **N. (%) country-seasons with 25-49 reported cases** | **N. (%) country-seasons with ≥ 50 reported cases** |
| --- | --- | --- | --- | --- | --- |
| 2016 | 13,974 | 29 | 16 (50.0%) | 5 (15.6%) | 11 (34.4%) |
| 2017 | 18,489 | 31 | 20 (45.4%) | 5 (11.4%) | 19 (43.2%) |
| 2018 | 15,230 | 21 | 24 (54.6%) | 2 (4.5%) | 18 (40.9%) |
| 2019 | 12,769 | 30 | 18 (46.2%) | 5 (12.8%) | 16 (41.0%) |
| 2020 | 5,129 | 13 | 20 (60.6%) | 6 (18.2%) | 7 (21.2%) |
| 2021 | 7,440 | 39 | 10 (38.5%) | 4 (15.4%) | 12 (46.1%) |
| 2022 | 22,062 | 76 | 11 (30.6%) | 4 (11.1%) | 21 (58.3%) |
| 2023 | 29,083 | 90 | 11 (26.8%) | 3 (7.3%) | 27 (65.9%) |
| 2024 | 23,824 | 95 | 14 (35.0%) | 3 (7.5%) | 23 (57.5%) |
| **Total** | **148,000** | **37** | **144 (43.0%)** | **37 (11.0%)** | **154 (46.0%)** |
