# Supplementary figures and images for: Global Epidemiology of Human Adenoviruses, 2016–2024: A Pre‐ and Post‐COVID‐19 Analysis of Circulation Patterns and Epidemic Timing
Source: Influenza Other Respir Viruses. 2026 Mar 4;20(3):e70236. doi: 10.1111/irv.70236 (PMC12959972; doi:10.1111/irv.70236)

# Argentina

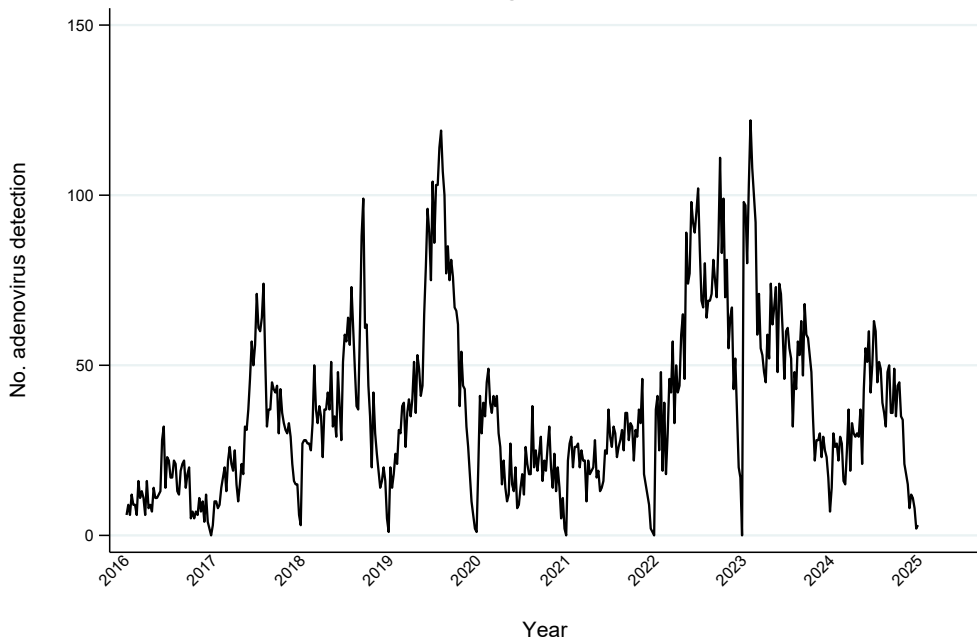

Supplement: Supplementary file 4 — Figure S4: Time series of circulation by country. [file IRV-20-e70236-s011.pdf]

# Australia

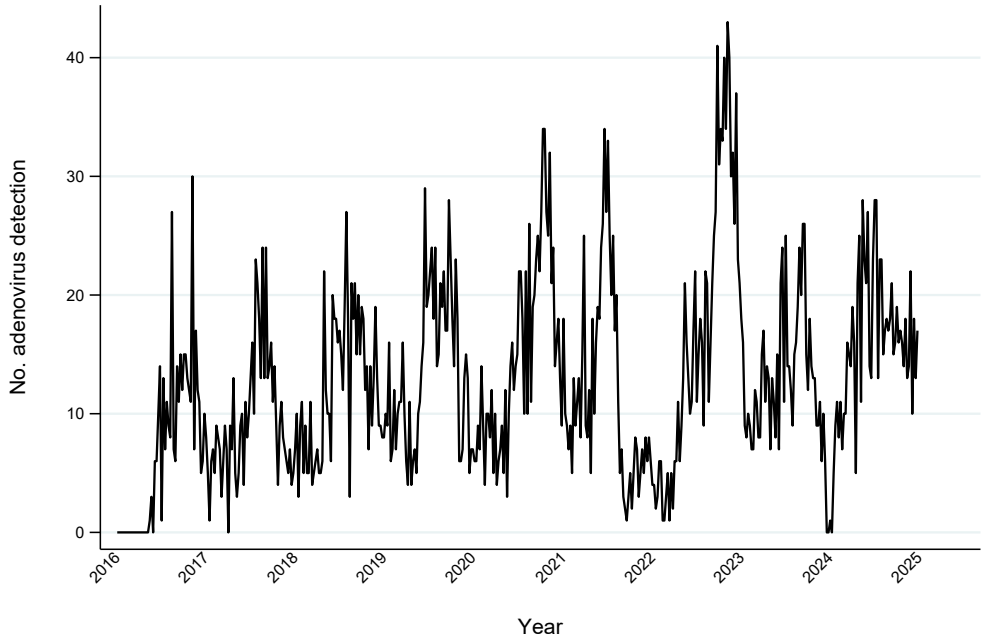

Supplement: Supplementary file 5 — Figure S5: Supporting information. [file IRV-20-e70236-s015.pdf]

# Brazil

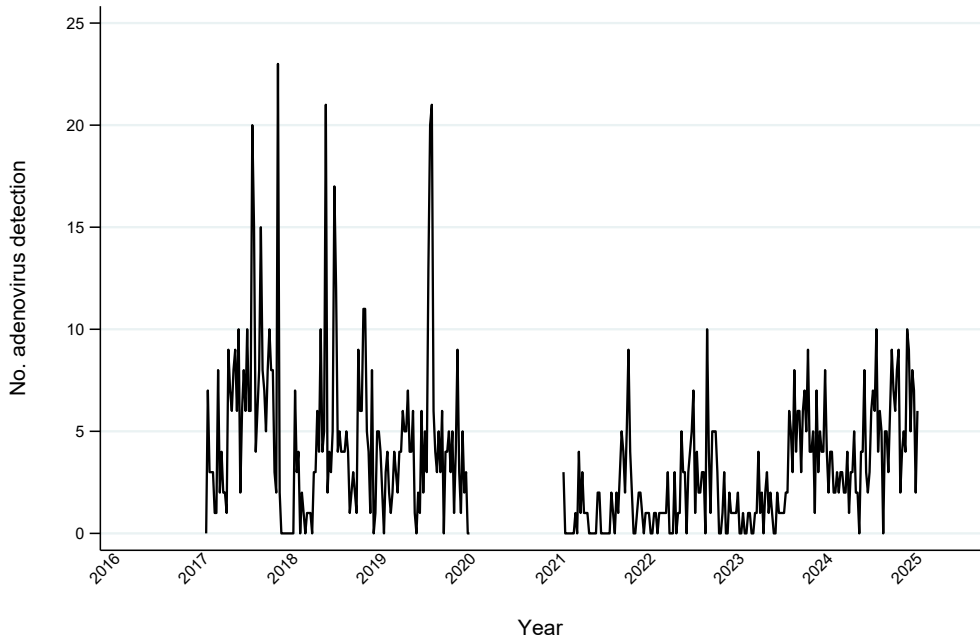

Supplement: Supplementary file 6 — Figure S6: Supporting information. [file IRV-20-e70236-s022.pdf]

# Canada

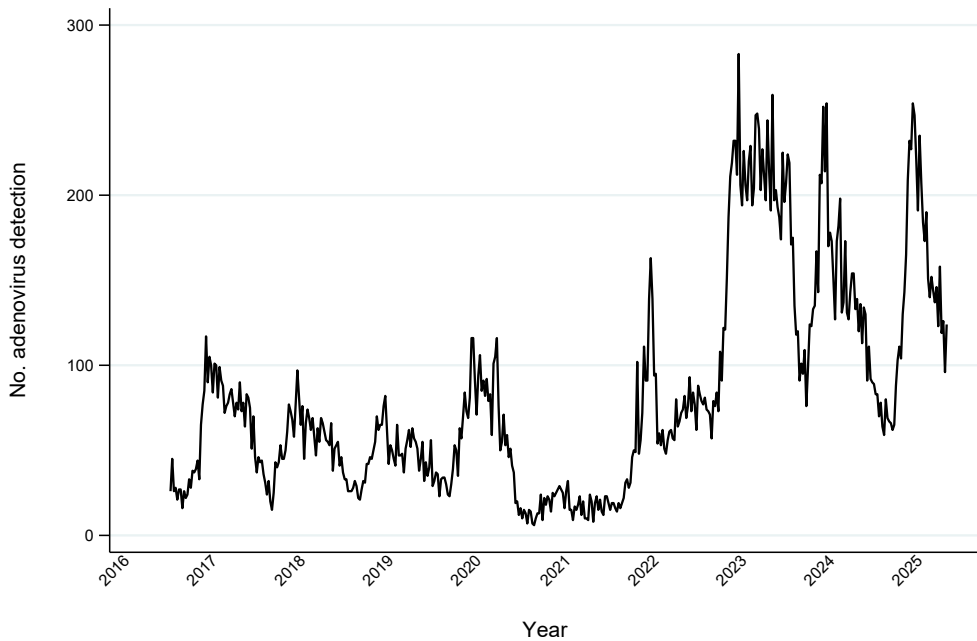

Supplement: Supplementary file 7 — Figure S7: Supporting information. [file IRV-20-e70236-s010.pdf]

# Chile

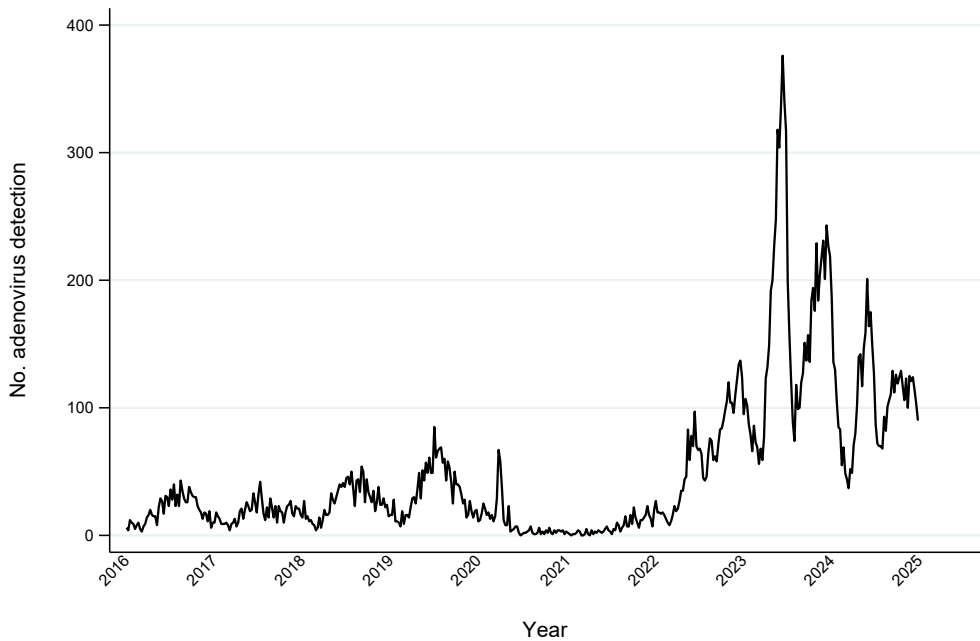

Supplement: Supplementary file 8 — Figure S8: Supporting information. [file IRV-20-e70236-s016.pdf]

# Colombia

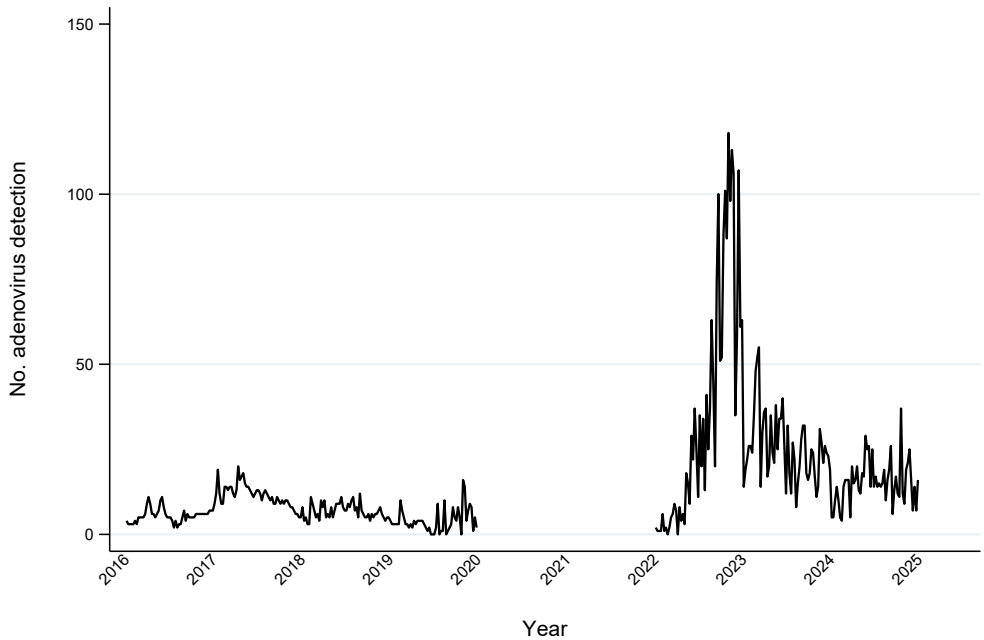

Supplement: Supplementary file 9 — Figure S9: Supporting information. [file IRV-20-e70236-s002.pdf]

# Dominican Republic

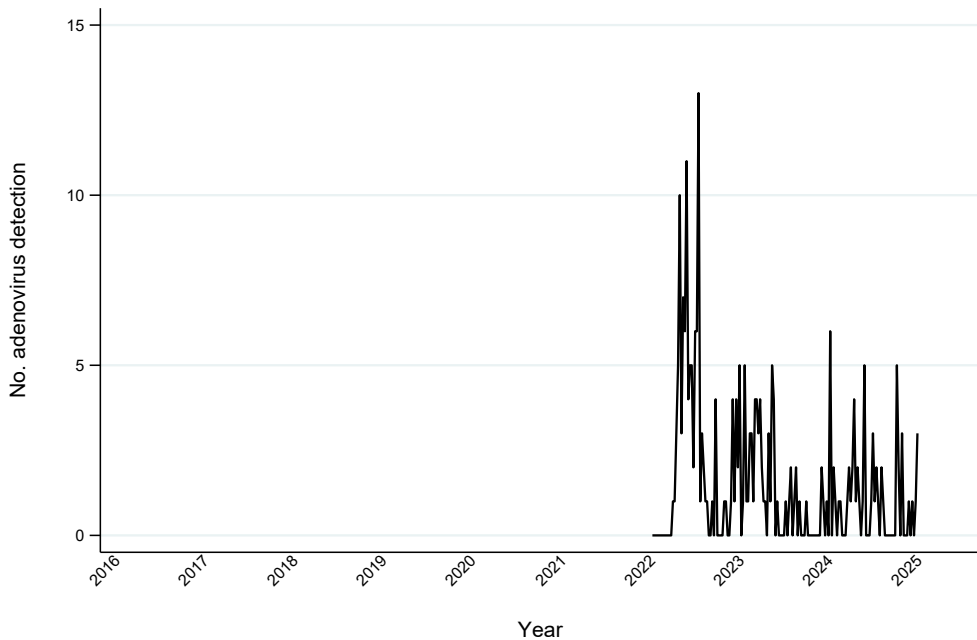

Supplement: Supplementary file 11 — Figure S11: Supporting information. [file IRV-20-e70236-s012.pdf]

# Guatemala

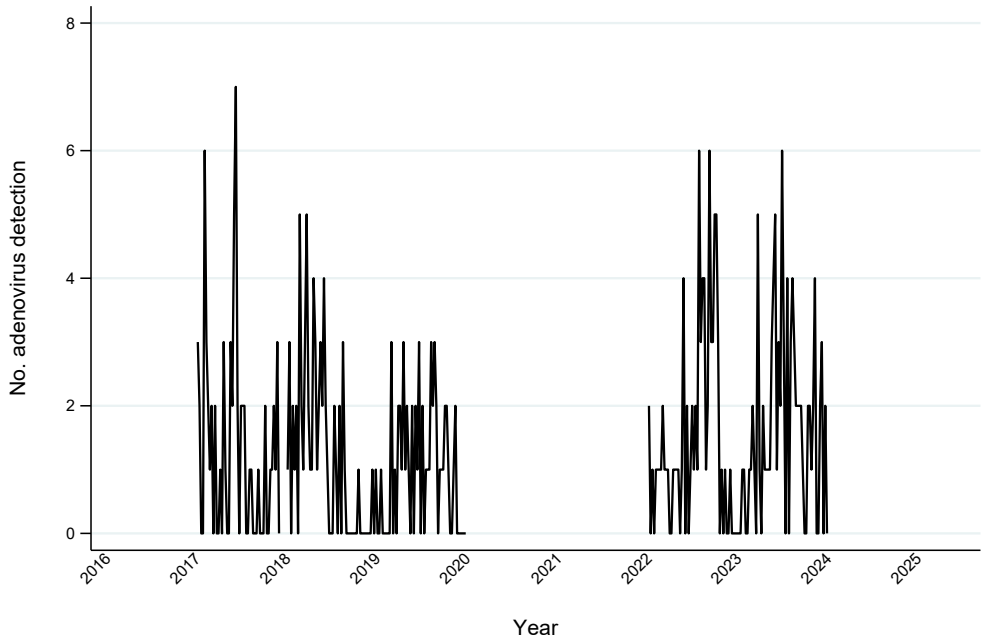

Supplement: Supplementary file 12 — Figure S12: Supporting information. [file IRV-20-e70236-s024.pdf]

# Hong Kong

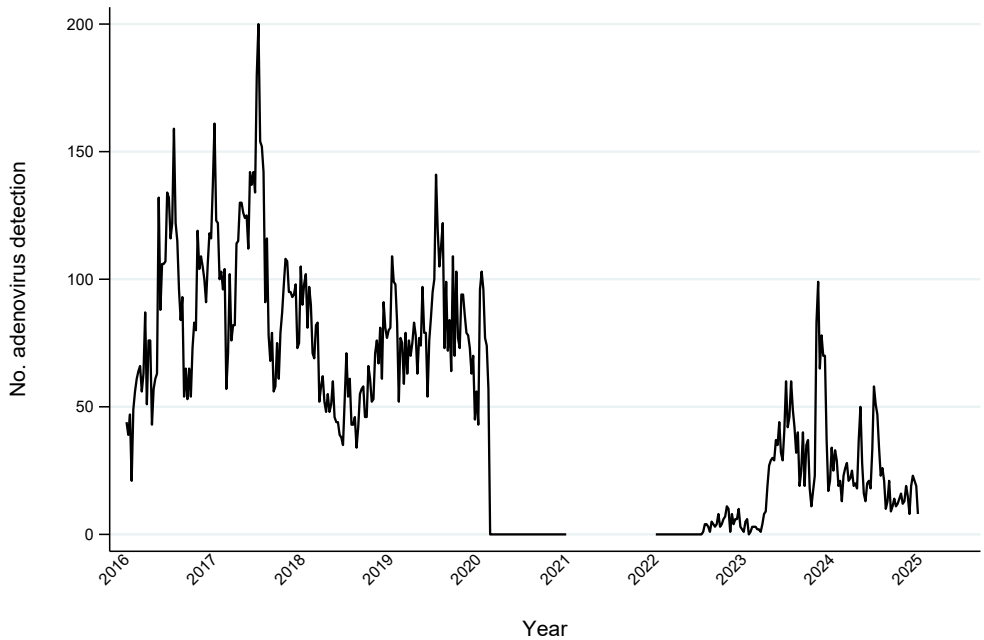

Supplement: Supplementary file 13 — Figure S13: Supporting information. [file IRV-20-e70236-s009.pdf]

# India

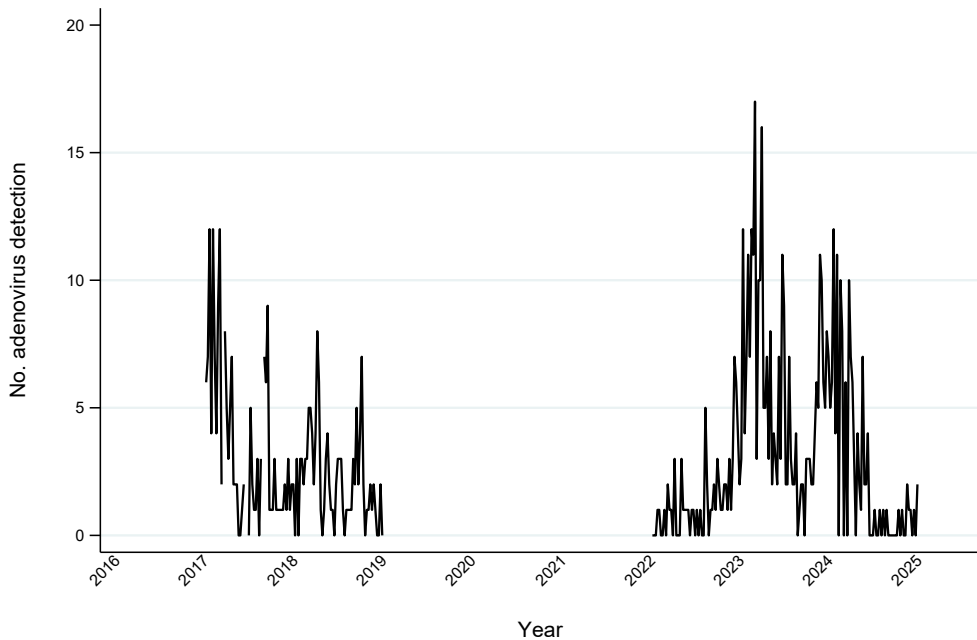

Supplement: Supplementary file 14 — Figure S14: Supporting information. [file IRV-20-e70236-s003.pdf]

# Japan

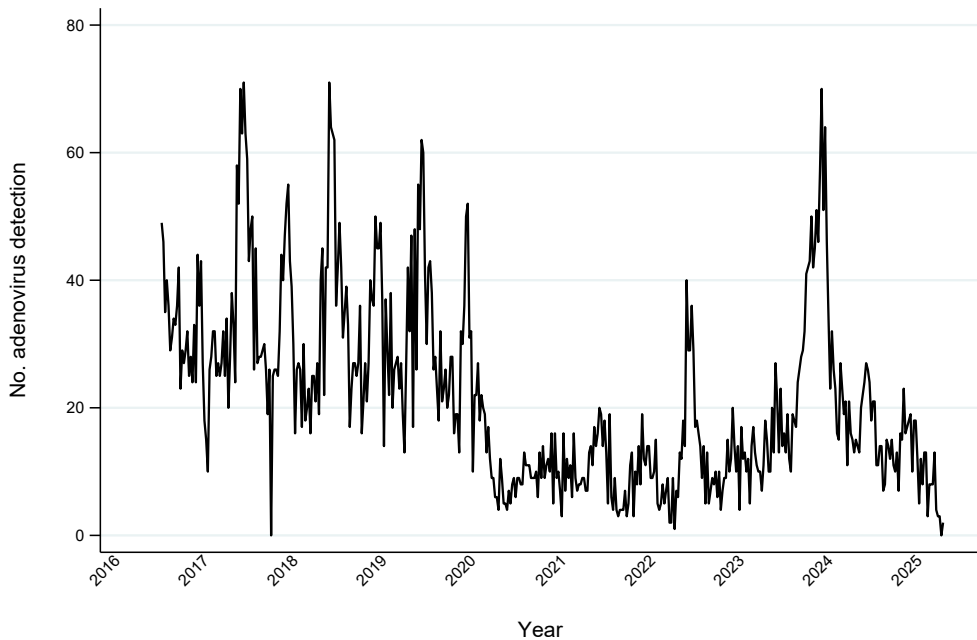

Supplement: Supplementary file 15 — Figure S15: Supporting information. [file IRV-20-e70236-s017.pdf]

# Malaysia

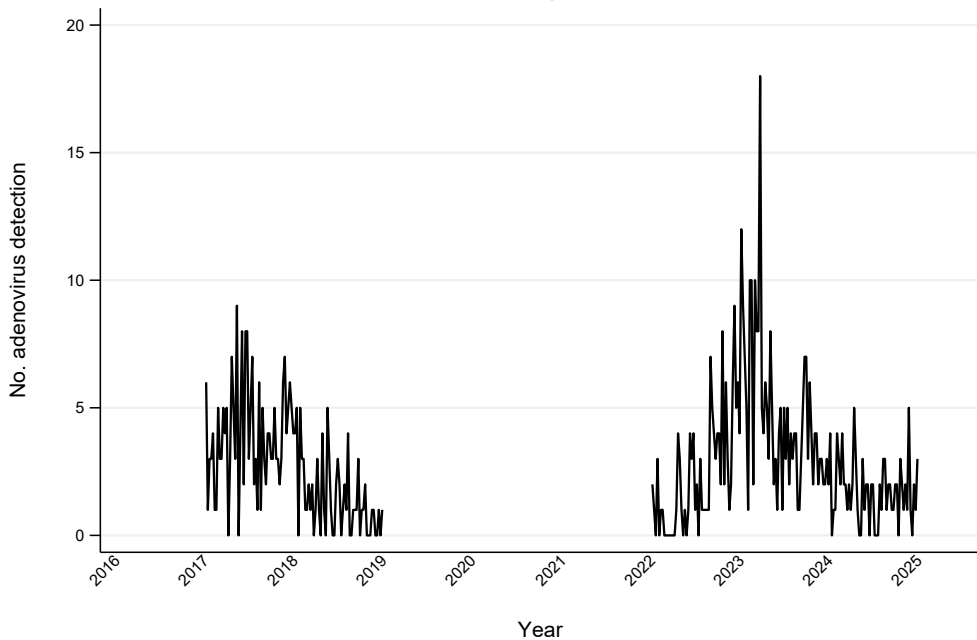

Supplement: Supplementary file 16 — Figure S16: Supporting information. [file IRV-20-e70236-s019.pdf]

# Mexico

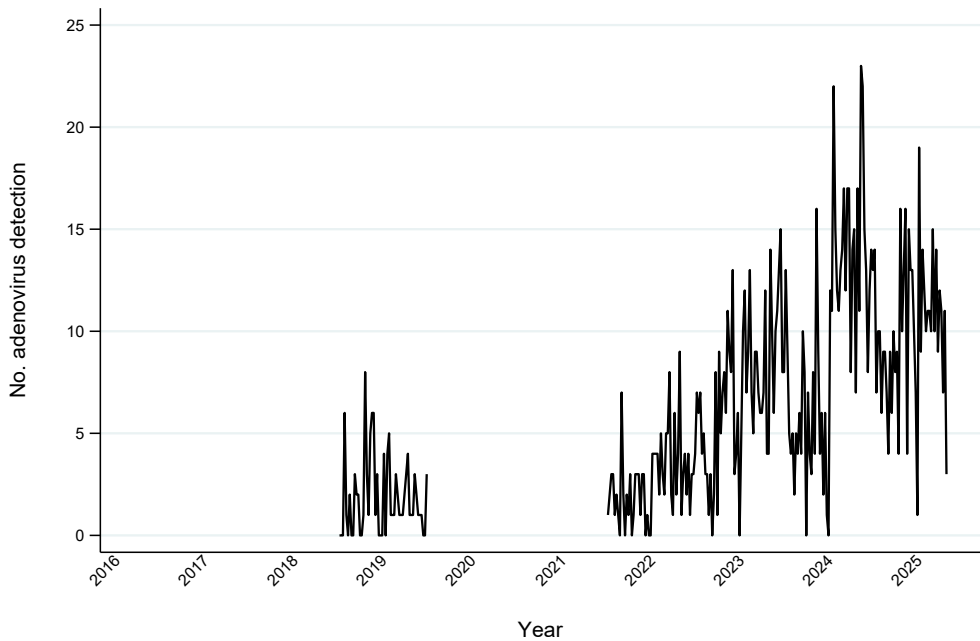

Supplement: Supplementary file 17 — Figure S17: Supporting information. [file IRV-20-e70236-s001.pdf]

# Mongolia

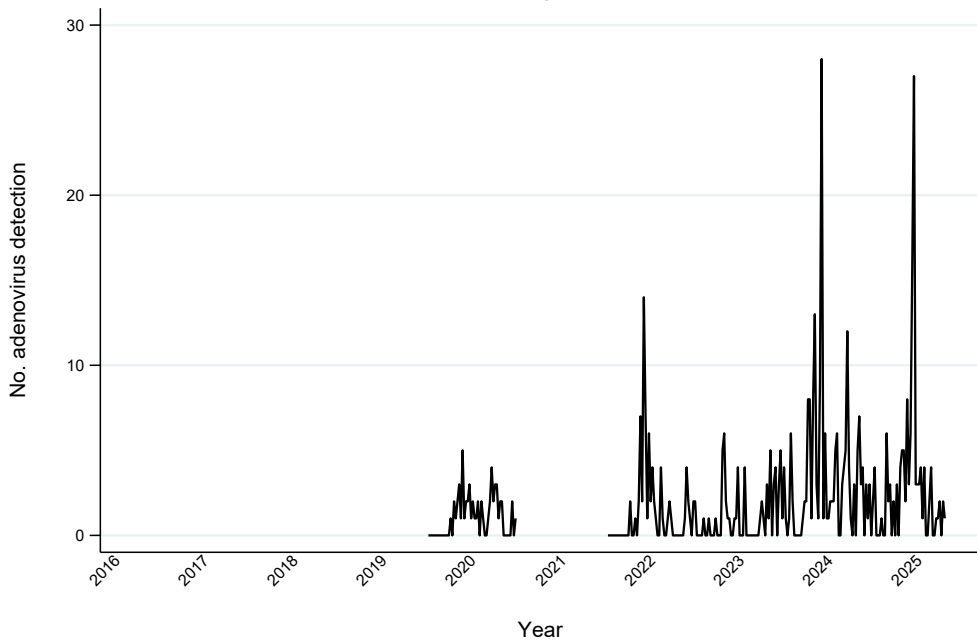

Supplement: Supplementary file 18 — Figure S18: Supporting information. [file IRV-20-e70236-s007.pdf]

# Oman

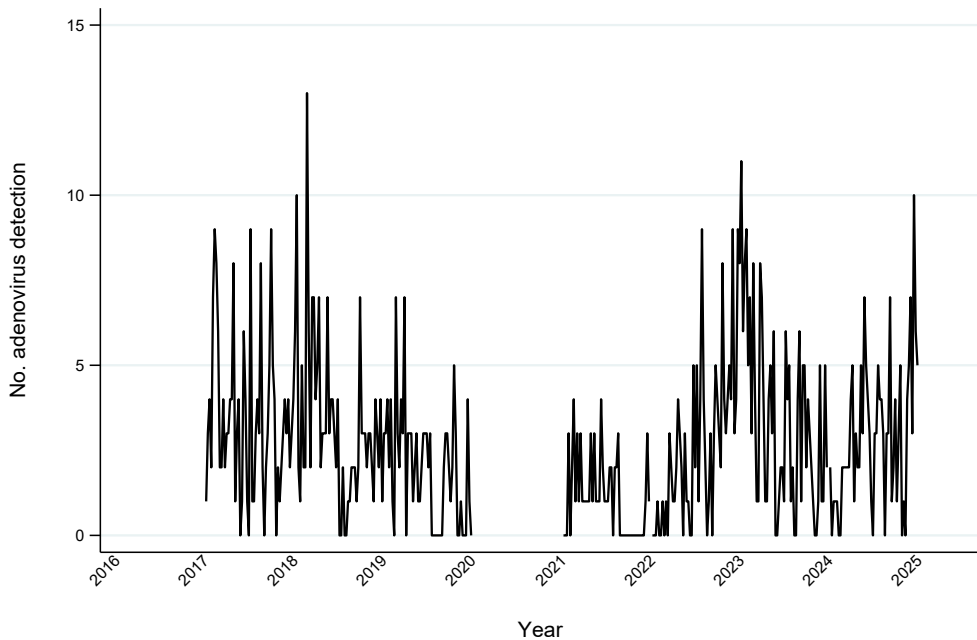

Supplement: Supplementary file 19 — Figure S19: Supporting information. [file IRV-20-e70236-s021.pdf]

# Panama

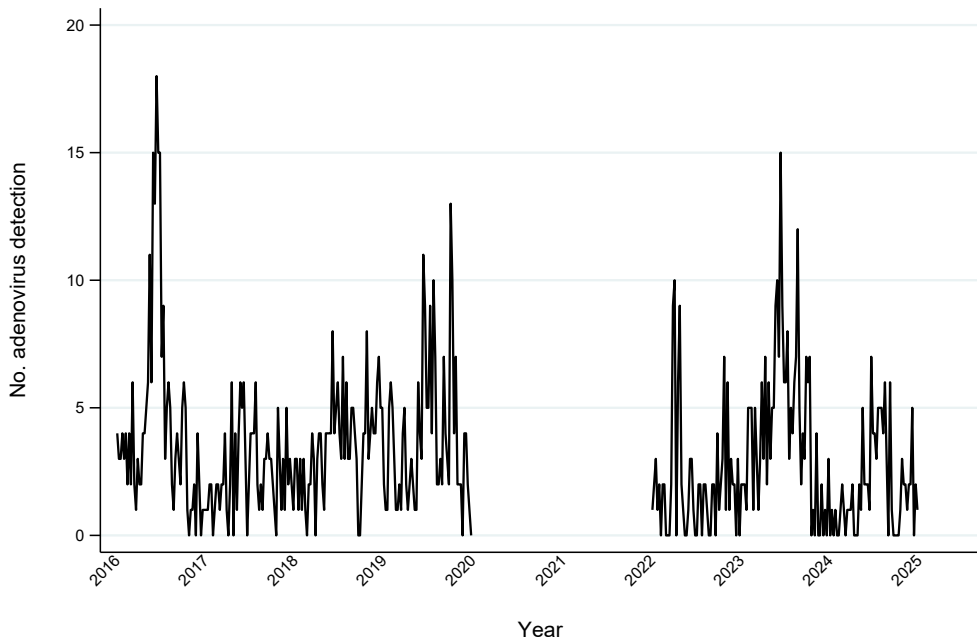

Supplement: Supplementary file 20 — Figure S20: Supporting information. [file IRV-20-e70236-s020.pdf]

# Paraguay

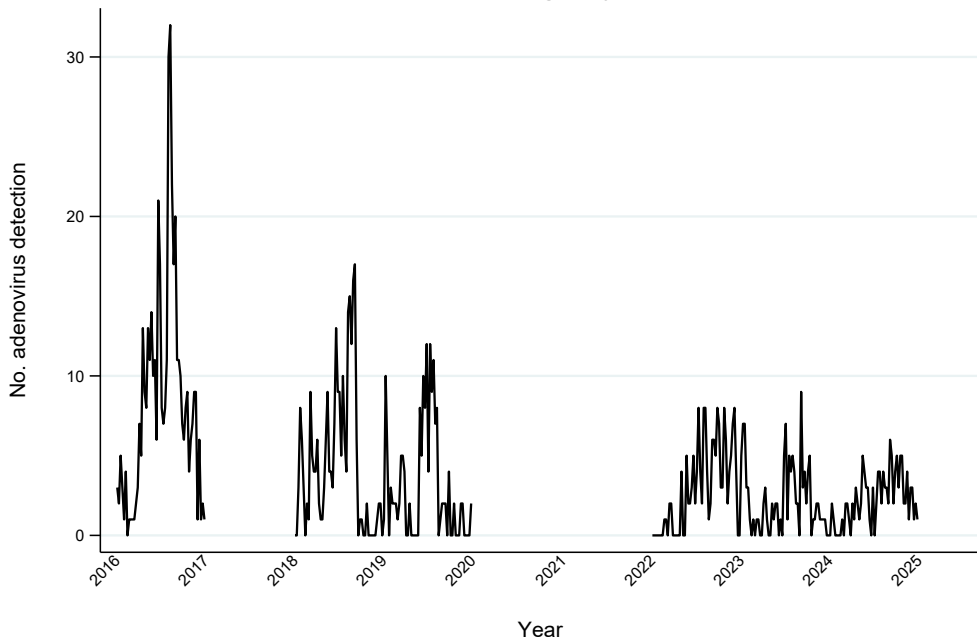

Supplement: Supplementary file 21 — Figure S21: Supporting information. [file IRV-20-e70236-s006.pdf]

# Qatar

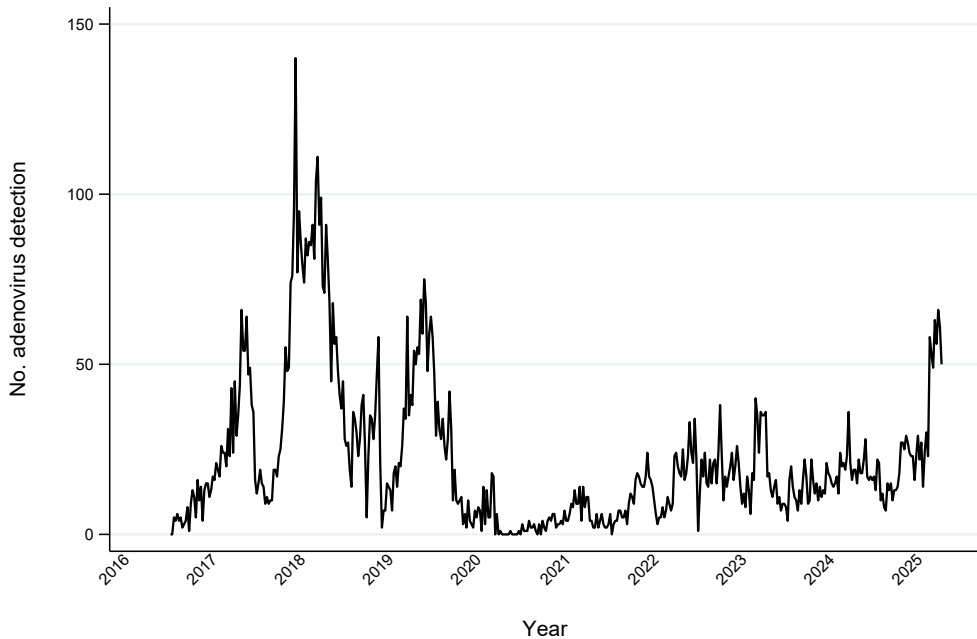

Supplement: Supplementary file 22 — Figure S22: Supporting information. [file IRV-20-e70236-s004.pdf]

# Thailand

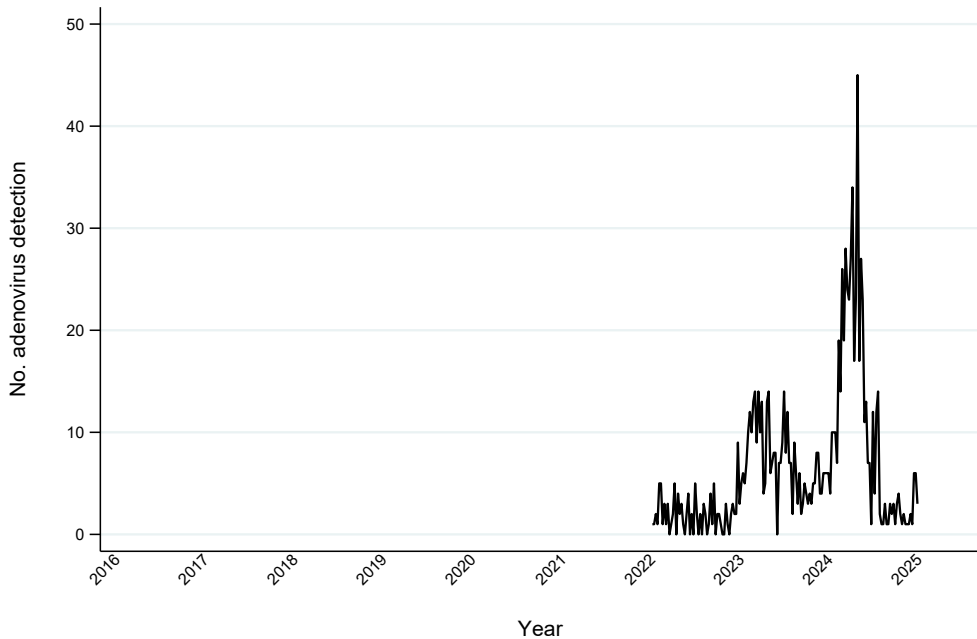

Supplement: Supplementary file 23 — Figure S23: Supporting information. [file IRV-20-e70236-s014.pdf]

# United Arab Emirates

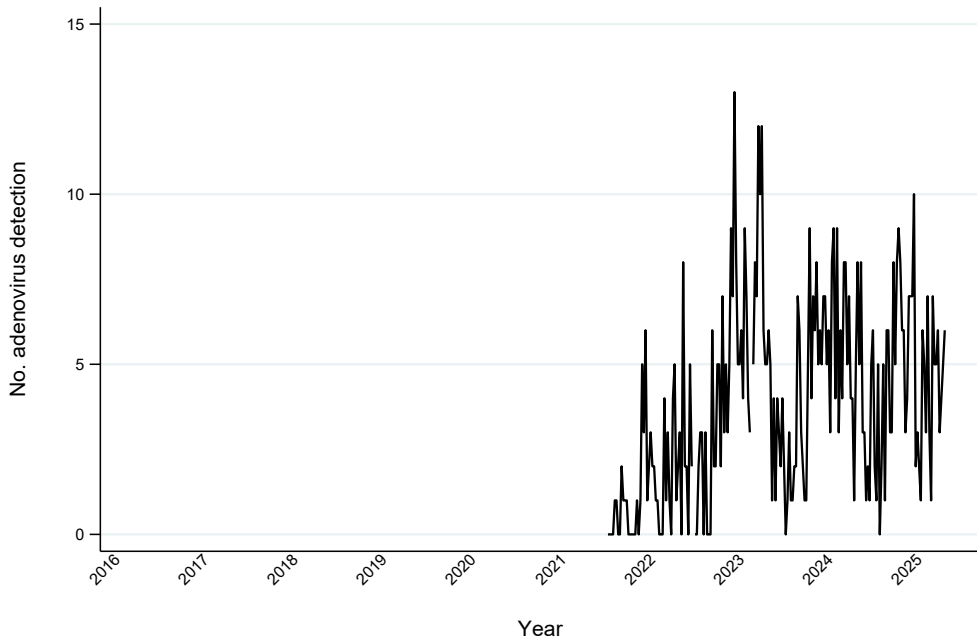

Supplement: Supplementary file 24 — Figure S24: Supporting information. [file IRV-20-e70236-s023.pdf]
