# Supplementary material for: Global Epidemiology of Human Adenoviruses, 2016–2024: A Pre‐ and Post‐COVID‐19 Analysis of Circulation Patterns and Epidemic Timing
Source: Influenza Other Respir Viruses. 2026 Mar 4;20(3):e70236. doi: 10.1111/irv.70236 (PMC12959972; doi:10.1111/irv.70236)
Supplement: Supplementary file 10 — Figure S10: Supporting information. [file IRV-20-e70236-s005.pdf]

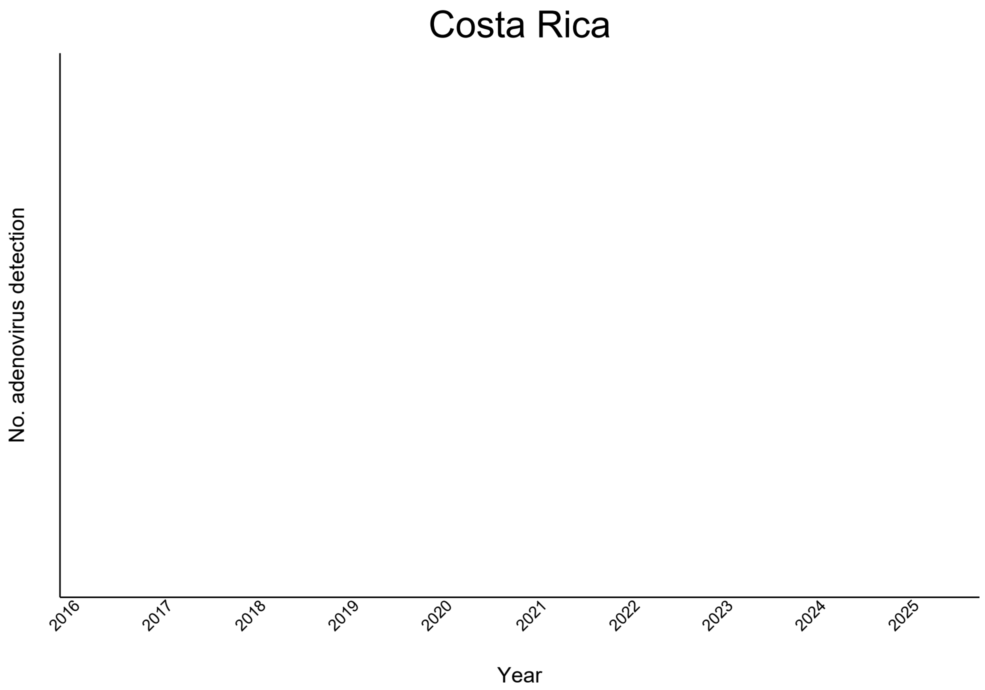
Costa Rica
No. adenovirus detection
2016
2017
2018
2019
2020
2021
2022
2023
2024
2025
Year
